# Successful Two-Stage Surgery with Preoperative Endoscopic Evaluation for Perforated Multiple Jejunal Diverticula in a Patient with Autosomal Dominant Polycystic Kidney Disease: A Case Report

**DOI:** 10.70352/scrj.cr.25-0308

**Published:** 2025-09-19

**Authors:** Ken Yonemitsu, Hiroaki Kasashima, Tatsunari Fukuoka, Mami Yoshii, Akihiro Tanaka, Shintaro Ozawa, Tsuyoshi Nishiyama, Yuki Seki, Masatsune Shibutani, Kiyoshi Maeda

**Affiliations:** 1 Department of Gastroenterological Surgery, Osaka Metropolitan University Graduate School of Medicine, Osaka, Osaka, Japan; 2 Department of Gastroenterological Surgery, Osaka City General Hospital, Osaka, Osaka, Japan

**Keywords:** autosomal dominant polycystic kidney disease, endoscopic evaluation, jejunal diverticula

## Abstract

**INTRODUCTION:**

Jejunal diverticulosis is a rare condition, often asymptomatic, but it can lead to serious complications such as diverticulitis or perforation. Management of perforated jejunal diverticula is challenging, particularly in patients with autosomal dominant polycystic kidney disease (ADPKD) who have undergone kidney transplantation and are receiving immunosuppressive therapy. Early diagnosis is often difficult due to nonspecific symptoms and the frequent presence of multiple diverticula, which increases the risk of postoperative complications.

**CASE PRESENTATION:**

We present the case of a 61-year-old woman with ADPKD, who developed jejunal diverticular perforation 3 years after undergoing ABO-incompatible kidney transplantation. She initially presented with mild abdominal pain and was managed conservatively, but her condition worsened 1 month later with evidence of free air on CT. Emergency surgery revealed multiple jejunal diverticula with a perforation on the mesenteric side, and a double-barrel stoma was created to avoid anastomotic leakage. Four months postoperatively, endoscopic and fluoroscopic evaluation from both the oral and stoma sides enabled accurate identification and marking of the diseased segment. A second-stage surgery was successfully performed with segmental jejunal resection and stoma closure. Histopathology confirmed multiple true diverticula, including at the perforation site. The patient recovered well and was discharged approximately 1.5 months later.

**CONCLUSIONS:**

This rare case of perforated multiple jejunal diverticula in a patient with ADPKD highlights the value of a two-stage surgical approach with preoperative endoscopic evaluation to enable safe and targeted resection in complex settings.

## Abbreviation

ADPKD
: autosomal dominant polycystic kidney disease

## INTRODUCTION

Autosomal dominant polycystic kidney disease (ADPKD) is a hereditary disorder characterized by the progressive development of renal cysts, often leading to end-stage renal disease.^1)^ Gastrointestinal manifestations of ADPKD are relatively uncommon, though colonic diverticulosis has been reported with increased frequency, particularly in post-transplant patients receiving immunosuppressive therapy.^2,3)^ By contrast, jejunal diverticulosis is a rare condition, typically asymptomatic but occasionally complicated by diverticulitis or perforation, which can lead to life-threatening peritonitis.

Management of jejunal diverticular perforation remains challenging due to its rarity, nonspecific clinical presentation, and the frequent coexistence of multiple diverticula, which increases the risk of postoperative complications such as anastomotic leakage.^4)^

Here, we report a rare case of perforated multiple jejunal diverticula in a patient with ADPKD who had previously undergone ABO-incompatible kidney transplantation. The patient was successfully treated with staged surgery, including initial stoma creation followed by partial jejunal resection and stoma closure, guided by preoperative endoscopic and fluoroscopic evaluation.

## CASE PRESENTATION

A 61-year-old woman with end-stage renal disease due to ADPKD had previously undergone ABO-incompatible kidney transplantation. Three years post-transplantation, she presented with mild abdominal pain. Abdominal CT revealed pneumatosis intestinalis without signs of perforation, and bowel perfusion was preserved. Conservative management with antibiotics was initiated. Although her condition subsequently showed a tendency toward improvement, mild abdominal pain and inflammatory response persisted. Additional evaluation, including repeat CT, was performed but revealed no findings that warranted surgery.

**Fig. 1:**
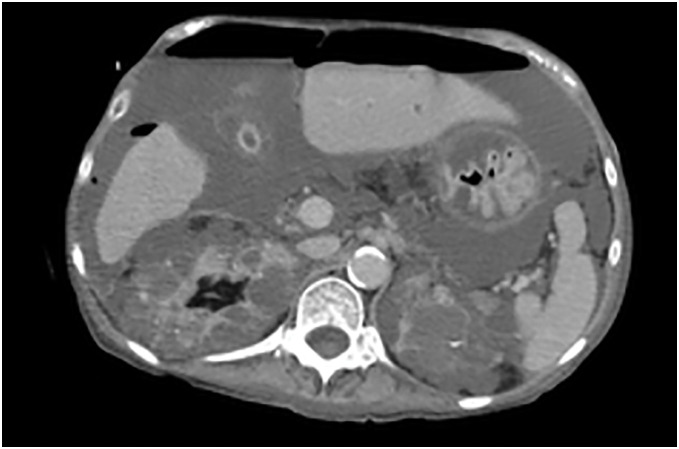
Free air was observed in the abdominal cavity on CT.

However, 1 month after admission, her abdominal pain worsened, and laboratory tests showed a marked inflammatory response (WBC 26,600/μL; CRP 14.41 mg/dL). Repeat CT revealed free air, raising strong suspicion for gastrointestinal perforation (**Fig. 1**), and emergency surgery was performed. Intraoperative findings revealed multiple diverticula on the mesenteric side of the jejunum, with a 1.5 cm perforation located approximately 10 cm distal to the ligament of Treitz. Given the extensive inflammation and presence of multiple diverticula, the risk of anastomotic leakage was deemed high, and a double-barrel stoma was created at the perforation site. Because the location carried a high risk of high-output stoma, concurrent creation of an enterostomy was considered. However, due to severe inflammatory changes and a long history of immunosuppressive and steroid use, the possibility of fistula formation failure at the same site was a concern; therefore, an enterostomy was not performed. Postoperatively, nutritional management was provided via total parenteral nutrition (TPN). Regular monitoring of hepatobiliary enzyme levels revealed no abnormalities, and clinically there were no symptoms of cholestasis such as pruritus or jaundice.

Early postoperative closure of the stoma was considered; however, the patient experienced repeated urinary tract infections and had poor general condition, so stoma closure was postponed until 4 months after the initial surgery.

**Fig. 2:**
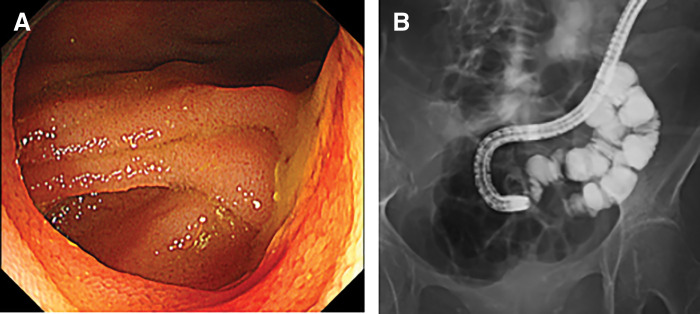
(**A**) Multiple jejunal diverticula were identified on endoscopic examination. (**B**) The extent of the diverticula was confirmed by fluoroscopic contrast study, and tattoo marking was performed.

**Fig. 3:**
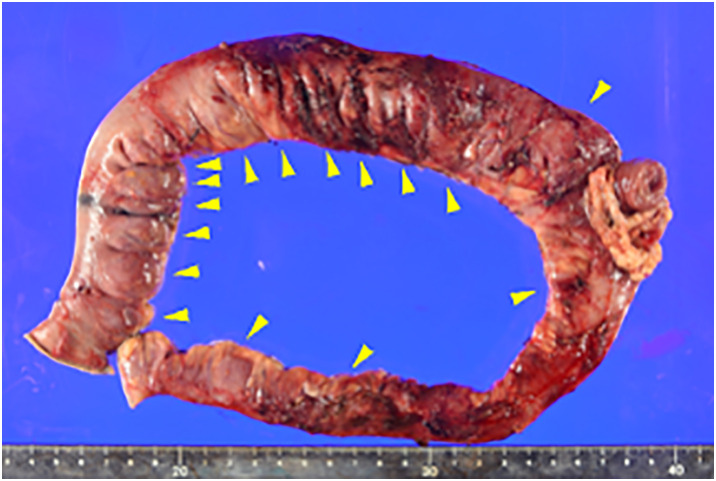
Multiple diverticula were observed on the mesenteric side of the resected specimen (arrowheads: diverticula).

Prior to stoma closure, both oral and stoma-side endoscopy were performed to identify the range of diverticulum. Multiple jejunal diverticula were identified, and their distribution was marked under fluoroscopic guidance (**Fig. 2A**, **2B**). Although severe adhesions due to the previous operation were encountered, the tattoo markings allowed for accurate identification of the affected segment. A partial jejunal resection including the diverticular segment was performed, followed by successful stoma closure (**Fig. 3**). Regarding the resection range, resection and anastomosis between diverticula was technically difficult because the jejunal diverticula were continuous, and it was also determined that removal of all diverticula would be unlikely to cause postoperative nutritional or absorptive disorders given the preserved bowel length. Therefore, approximately 50 cm of jejunum was resected according to the preoperative markings.

**Fig. 4:**
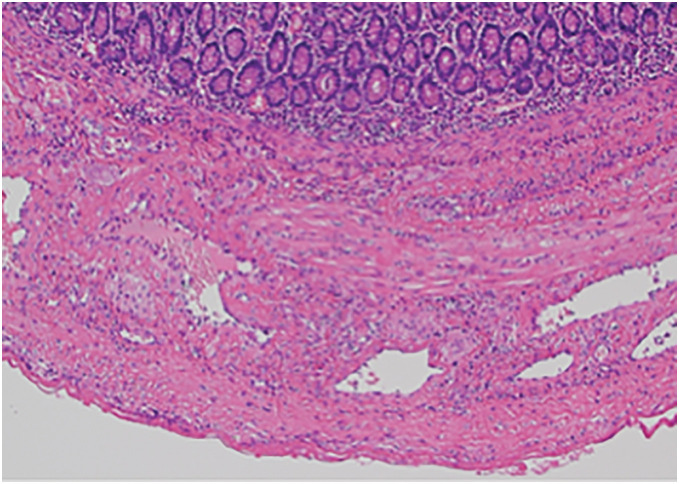
Histopathological examination revealed numerous true diverticula.

Histopathological examination revealed multiple true diverticula containing smooth muscle on the mesenteric side of the jejunum, including at the site of perforation (**Fig. 4**). The postoperative course was favorable, with only mild paralytic ileus and a urinary tract infection, and the patient was discharged approximately 1.5 months after surgery.

## DISCUSSION

Jejuno-ileal diverticulosis was first described by Sommering in 1794. The prevalence of small intestinal diverticula ranges from 0.06% to 1.3%.^5)^ Most patients are in the 6th and 7th decades of life. Small bowel diverticula is twice as frequent in men than in women. The diverticula have a tendency to be smaller and fewer as one progresses distally in the small bowel. Diverticular disease is more common in the proximal jejunum (75%), followed by the distal jejunum (20%) and the ileum (5%).^6)^

While colonic diverticulosis is frequently observed in patients with ADPKD,^7–9)^ reports of small intestinal diverticulosis in this population are limited to case reports, and the underlying pathogenesis remains poorly understood. Nevertheless, 30%–60% of patients with small intestinal diverticula are also reported to have colonic diverticulosis.^10,11)^ This overlap, along with the presence of jejunal diverticula in some ADPKD patients, suggests a possible but yet unproven association between the two conditions.

Most small intestinal diverticula are asymptomatic, and only about 20% of patients develop clinical symptoms.^4)^ Complications include diverticulitis, perforation, and bowel obstruction, with perforation typically resulting from underlying inflammation.^12)^ Small bowel diverticula, whether true or false, overwhelmingly tend to occur on the mesenteric attachment side,^13)^.which often makes them difficult to detect intraoperatively from the serosal surface. When perforation occurs, inflammation may remain localized within the mesentery, forming abscesses that adhere to adjacent structures. As a result, peritoneal irritation signs may be mild or delayed, complicating early diagnosis. This diagnostic delay contributes to a reported mortality rate of approximately 24% for small bowel perforation,^14)^ highlighting the importance of prompt recognition and timely surgical intervention.

In the present case, although small amounts of free air were detected on imaging at the time of presentation, the patient’s symptoms were mild, making early diagnosis and treatment decisions difficult. As her clinical condition deteriorated, emergent surgery was performed based on imaging findings and laboratory data.

Surgical resection of the affected bowel segment is the standard treatment for perforated small bowel diverticula.^12)^ However, surgical strategy must be individualized based on the extent of diverticular involvement, presence of complications, and the patient’s overall condition. In our case, due to the presence of multiple diverticula and surrounding inflammation, a double-barrel stoma was initially created to reduce the risk of anastomotic failure.

Preoperative endoscopic evaluation prior to stoma closure is considered a useful approach for assessing the extent of disease in staged surgery. In this case, endoscopy—performed both orally and through the stoma—allowed accurate identification of the diverticular segment under fluoroscopic guidance. This enabled precise planning of the resection extent and contributed to a favorable postoperative outcome. Regarding the resection range, although there are reports suggesting that extensive resection of non-inflamed diverticula is undesirable from the perspective of preserving small bowel function,^12)^ in this case, histopathological findings revealed mild inflammatory changes in multiple diverticula apart from the perforated site. Moreover, given that the resected bowel length was unlikely to cause postoperative nutritional or absorptive impairment, we considered that resection with a preventive intent was reasonably justified.

On the contrary, although the exact mechanism of gastrointestinal diverticulum formation in ADPKD has not been clearly elucidated, possible contributing factors include structural fragility of the intestinal wall, abnormalities of the smooth muscle, disorders of collagen metabolism, and decreased intestinal resistance due to immunosuppressive therapy.^6,12,13)^ Therefore, in the present case as well, it cannot be ruled out that new diverticula may form in the remaining small intestine in the future, and we believe that long-term follow-up is warranted.

## CONCLUSIONS

We experienced a rare case of jejunal diverticular perforation in a patient with ADPKD after kidney transplantation. Staged surgery with preoperative endoscopic evaluation allowed for appropriate resection and a favorable outcome.
